# Technology in care systems: Displacing, reshaping, reinstating or degrading roles?

**DOI:** 10.1111/ntwe.12229

**Published:** 2022-02-06

**Authors:** Kate A. Hamblin

**Affiliations:** ^1^ Centre for International Research on Care, Labour and Equalities (CIRCLE), Faculty of Social Sciences University of Sheffield Sheffield UK

**Keywords:** automation, care work, digital technology, internet of things, job quality, robotics, smart devices, social care, telecare, UK

## Abstract

In the United Kingdom and further afield, policy discourse has focused on the efficiencies technology will afford the care sector by increasing workforce capacity at a time when there are recruitment and retention issues. Previous research has explored the impact of telecare and other technologies on roles within the care sector, but issues related to job quality and the consequences of newer digital technologies that are increasingly being deployed in care settings are under researched. Through an exploration of the literature on robotics and empirical studies of telecare and mainstream ‘smart’ digital technology use in UK adult social care, this paper examines how these technologies are generating new forms of work and their implications for job quality, arguing the tendency to prioritise technology results in the creation ‘machine babysitters’ and ‘fauxtomatons’.

## INTRODUCTION

In our everyday lives, technology is ubiquitous. Özkiziltan and Hassel (2020) highlight that many jobs are automated to make them safer and their outputs more uniform, whilst in our homes, temperature, lighting and entertainment systems are 'smart' and robots have taken on household tasks, mowing lawns, vacuuming and clearing gutters. Technologies that support the care of older and disabled people, a longstanding feature of care systems, increasingly include digital devices and systems. The use of technologies in UK adult social care has been the focus of policy rhetoric and investment as a ‘silver bullet’ (Eccles, 2020) for a system described as ‘in crisis’ due to factors including an ageing population, a decline in resources in real terms and workforce recruitment and retention issues (Hamblin, 2020a). Successive Secretaries of State for Health and Social Care have repeatedly described technology as ‘transformative’ (Hancock, 2019; Javid, 2021) in relation to care quality and its ability to ‘free up time’ of the care workforce (DHSC, 2021, p. 42). In 25 years, there has been in excess of 25 UK government and official reports advocating the use of technology in care (Barlow et al., 2012) and significant funding opportunities, with a focus in the 2000s on ‘telecare’^1^ systems and a more recent shift to digital technologies, such as mainstream ‘Internet of Things’ devices, artificial intelligence, data analytics and newer assistive devices, including robotics (Wright, 2020). The 2021 White Paper *People at the Heart of Care* (DHSC, 2021) proposed an additional £150million to ‘drive greater adoption of technology and achieve widespread digitisation’ (p. 7) with focused action around providers of care.

While studies of care and technology have examined the impact on those receiving support (Hamblin, 2017; Lynch et al., 2019), to date the implications for those *providing* paid care work have not been as widely discussed. Though technology has been presented as a substitute for routine aspects of care work and as a means to manage costs, it also creates new care tasks and jobs. The *quality* of both the roles altered and created is crucial if technology is to address the challenges facing social care, as workforce recruitment and retention are key pieces of that puzzle. This paper considers the implications of technology on paid work within care systems, focusing on two under‐explored areas: its *impact on pre‐existing jobs*, including replacing and reshaping care worker roles and tasks; and its potential to *create new* ‘good’ *jobs*. We draw on literature related to automation and its impact on work, which characterises technology as replacing, reshaping and reinstating job roles. The paper is also influenced by Science and Technology Studies (STS), highlighting that technology is social and political. Taking robotics as an example, we first explore the impact of these technologies on existing care worker roles, before we use two qualitative, mixed‐methods empirical studies of technology and care to address the type and quality of roles created. In addressing these issues, the paper provides a critique of simplistic policy narratives which present technology as a solution to workforce and resource shortages in UK adult social care.

### Automation, work and ‘good jobs’

In the early 1970s, new digital technologies began to rapidly accelerate the automation of many previously labour‐intensive tasks and jobs (Özkiziltan & Hassel, 2020). However, the presentation of labour's replacement by automation as inevitable has been critiqued as ‘technological determinism’ that misses its ‘contingent, complex and unintended outcomes […] some jobs become obsolete and are displaced, but new ones are created and yet others incorporate elements of technologies that may be transformative’ (Howcroft & Taylor, 2014, p. 2). Developments in technology may actually increase the number of jobs available, due to rising demand and ‘spill over effects’ (Gregory et al., 2016), and will also alter existing jobs, increasing the level of skill required as technology undertakes the more routine aspects of some roles (Özkiziltan & Hassel, 2020). Business consulting groups with particular interests are argued to have heavily influenced the policy discourse on the ‘Future of Work’ and automation, presenting the solution to the ‘Machine v. Human’ challenge as the ‘upskilling’ of the latter to accommodate the former (Schlogl et al., 2021).^2^

Acemoglu and Restrepo's (2019) framework encapsulates these debates, highlighting three effects of automation. Though they argue there are *displacement* effects, whereby machines replace human labour, there are also *productivity* effects, where demand for non‐routine, non‐automated tasks, including personal care, increases, and *reinstatement* effects, creating new jobs that humans perform better than machines, such as the design, operation and maintenance of the new technologies introduced. This paper considers both how far technologies are reshaping or displacing existing care worker roles, and the extent to which ‘reinstatement’ is occurring through the creation of new job roles within care systems. The first of our research questions is therefore: *(1) Does technology have displacement, productivity or reinstatement effects in the UK adult social care sector?* that is, is technology (a) replacing care workers; (b) executing the more routine and mundane tasks care workers perform; and/or (c) creating new roles within the care sector?

This paper addresses a second question regarding job quality, which Acemoglu and Restrepo's (2019) framework does not explore, but is a highly relevant issue and the focus of both academic and policy debates (Dent, 2021; Taylor et al., 2017). Some authors claim that far from creating a shortage of jobs, technology and automation will increase the number available, albeit with negative effects on job quality as ‘the key story […] is how lower paid and lower quality work is reproduced, and potentially entrenched, alongside technological progress’ (Spencer & Slater, 2020, p. 118). In a period marked by economic downturns, austerity, the rise of the ‘gig economy’ and precarious employment, ‘good jobs’, ‘quality jobs’ and ‘decent work’ are pertinent issues that are characterised as multidimensional and complex (Adamson & Roper, 2019). Various reviews of the literature have identified the following elements of ‘quality’ and ‘good jobs’: (1) health and safety; (2) job content and characteristics; (3) pay and rewards; (4) terms of employment, job security and advancement; (5) scope for autonomy; (6) work–life balance and manageable workloads; (7) representation and social dialogue; and (8) workforce relations (Clarke, 2015; Warhurst et al., 2017). These elements align with the International Labour Organisation's (2013) definition of ‘decent work’, which has been built on to create the ‘5R Framework for Decent Care Work’ that seeks to promote ‘more and decent work for care workers’ by: Recognising, Reducing and Redistributing unpaid care work; Rewarding (more and decent work for care workers); and Representation (social dialogue and collective bargaining for care workers; Addati et al., 2018, p. xliii).

The care sector faces recruitment and retention difficulties, exacerbated by the UK's withdrawal from the European Union and the COVID‐19 pandemic (Turnpenny & Hussein, 2021). Care work is ‘characterised as ‘dirty’ work, comprising low‐quality, low‐skilled and poorly rewarded jobs (Addati et al., 2018), influenced by a reliance on ‘time‐to‐task’ commissioned care models, stress and ‘emotional labour’ (Turnpenny & Hussein, 2021) and associated with the gendered division of labour (Hansen, 2016; Twigg et al., 2011). Studies have however highlighted positive elements that attract workers to the care sector and lead many to stay, including its ‘valuable and self‐affirming’ role in society (Clarke, 2015, p. 202) and scope for skills development, autonomy and dignity (Stacey, 2005). However, processes can lead to the ‘degradation of work’, and turn ‘good’ jobs ‘bad’ (Braverman, 1974). It is important to understand whether technologies used in the care sector alter the content of care worker roles positively by taking on routine tasks or negatively, ‘degrading’ these jobs by removing emotionally rewarding aspects. Equally, if there is a reinstatement effect, are the new roles created by technology in the care sector ‘good’ quality jobs? The second question is therefore: *(2) Does the introduction of technology have implications for the quality of jobs in the care sector?*

The subject of the paper was inspired, in part, by comic artist Krish Raghav's^3^ series, ‘Bullshit Jobs’ where ‘machine babysitters’ hand out machine‐produced queue numbers to guests and use a touch‐screen on their behalf: ‘our companies bought big shiny machines “to improve service efficiency” but they don't actually trust people to use them properly. So we babysit them. Feed them paper at mealtimes. Turn them off and on again’. As the cartoon ends, Raghav wonders, ‘Are we serving the machines or people? Somehow both, somehow neither’. Raghav's series introduced another concept related to technology and the creation of new roles which is the main focus of this paper: ‘fauxtomatons’. In another cartoon, a Shared Bike redistributor collects and returns bikes to docking stations for app users to collect and notes: ‘People attribute my work to the magic of an “app” but these bikes don't teleport themselves… I guess I'm just a “fauxtamaton”—invisible labor that keeps of the façade of shiny technological efficiency’; similarly, the roles technology creates or, to use Acemoglu and Restrepo's (2019) framework, ‘reinstates’ in the care sector may be invisible, providing care on behalf of the seemingly efficient technology.

Other authors have taken a critical approach to recent advances in automation, with Taylor (2018, 2019) coining the phrases ‘faux‐bot revolution’ and ‘fauxtomation’ to describe how ‘[a]utomation is mostly a charade—a ploy by firms to look sophisticated while humans continue to do grunt work behind the scenes’ as well exploring whose interests this charade serves, noting ‘[t]he phrase “robots are taking our jobs” gives technology agency it doesn't (yet?) possess, whereas “capitalists are making targeted investments in robots designed to weaken and replace human workers so they can get even richer” is less catchy but more accurate’. Sadowski (2018) similarly shares examples of ‘Potemkin AI’ (taking its name from the Russian minister who fabricated fake villages to obscure the real state of affairs from Empress Catherine II), where ‘sophisticated’ technologies endowed with ‘objectivity, neutrality, authority, efficiency’ are in actuality powered by humans. Sadowski argues the ‘black box’ which obscures technology's mechanical inner workings now includes the human labour behind seemingly autonomous systems, but obfuscation has been edged towards deception motivated by power and profit, with the illusion used to hide the exploitation of human labour.

To explore these issues in more detail and in relation to social care specifically, we first present examples from the academic literature regarding how care worker roles are altered by the use of robotics, before outlining the methodologies used in two studies of technologies (telecare and mainstream ‘smart’ digital devices) in UK adult social care. To reflect its use in policy discourse where ‘technology’ is presented as a single entity and a solution to challenges facing care systems (Neven, 2015; Pols & Willems, 2011), we have thus far used the term in a monolithic way, eliding the diversity of devices and systems deployed in UK adult social care. However, drawing on STS, we acknowledge that technology in care is a ‘complex intervention’ (Hamblin et al., 2017) whose ‘benefits might best be summarised as applicable to some people, in some circumstances, at some points in their lives. In short, it has been less of a panacea than policy agendas initially suggested’ (Eccles, 2020, p. 3). Eccles argues policy discourse's presentation of a ‘technological fix’ as a ‘silver bullet’ for social care's issues wrongly assumes that technology functions separately from social relations. Instead technology and care are coproduced as the former is reliant on people to function who may ‘tame’ and ‘tinker’ with devices while at the same time, these devices are accompanied by ‘scripts’ and ‘directives’ which shape both care and care work, inscribed with the particular politics of their designers, developers and investors (Pols & Willems, 2011). Technology is therefore never asocial or apolitical. We acknowledge there are a broader range of technologies being deployed in care and myriad ways they may affect care relationships, roles and tasks, but focus on specific technologies as they represent the past and status quo of much of what is commissioned in UK adult social care (telecare), the emerging present (mainstream devices) and the purported future (robotics), with the aim to illuminate the wider context of technologically enabled care work.

## ‘MACHINE BABYSITTERS’ AND CARE ROBOTS

Robotics^4^ has become a burgeoning area of interest as a means to increase care system capacity and efficiency (Cruickshank & Trim, 2019; Prescott & Caleb‐Solly, 2017). To date, evidence about the use of robotics in social care is limited to small‐scale pilots and trials, with little practical application in UK adult social care (POSTnote, 2018; Wright, 2021b). Whereas ethical concerns have been raised about the implications for people needing care, including how robotics might reduce social contact, privacy and liberty (Sharkey & Sharkey, 2012; Sparrow, 2016), the moral considerations for the care workforce are under researched (Consilium Research & Consultancy, 2018). Though Coeckelbergh (2010) theorised that robots could undertake mundane and routine care tasks, thereby increasing care workers' capacity to provide emotionally fulfilling support—the productivity effect (Acemoglu & Restrepo, 2019)—empirical studies suggest the ‘machine babysitter’ concept is apt.

Hasse (2013) explored the impact of Paro, a socially assistive robot (SAR) in the form of a baby harp seal, on the professional identities of residential care workers through the ways the device altered their activities. Hasse noted Paro is marketed and deployed as a means to calm and soothe residents, reallocating this important and valued aspect of care workers' roles in order that the care setting appeared ‘innovative’ through its use of robots. At the same time, Paro necessitated extra work for staff and schedules were drawn up to accommodate this. Existing care tasks were therefore reconfigured as staff felt the emotional, human‐to‐human aspects of care workers' roles could be perceived as ‘old fashioned’ once Paro was introduced to the setting.

Wright's (2019) ethnographic study of the use of robots, including Paro, in Japanese residential care facilities found that rather than replacing care workers, these devices merely *displaced* them by conducting some of the more ‘hands on’ tasks care workers would have undertaken while at the same time, creating new demands, ‘increasing the amount of work tasks for human caregivers, deskilling aspects of care labor, and raising overall costs’ (ibid: 331). These additional work tasks were less visible and not focused on the care for residents. The introduction of Paro required care workers to become in effect ‘machine babysitters’, with Wright citing an example of one resident learning how to remove the robot's faux‐fur skin and as an expensive investment, staff had to intervene to ‘protect’ Paro.

Wright (ibid.) also observed another SAR—Pepper—in use and the degradation of work was again apparent. Although Pepper is marketed as a ‘standalone’ robot which can be left to function on its own and therefore replace care workers, in practice a member of staff always needed to ‘babysit’ it. Indeed, Wright describes how care workers used similar language to describe their roles in relation to Pepper as with the people they cared for—Pepper was ‘hard of hearing’ and needed help communicating, was at risk of falling and ‘injuring’ itself and therefore needed staff to ‘watch and protect’ it. Thus ‘[t]his extra human labor has been hidden in plain sight, discounted in promotional videos, and overlooked in enthusiastic state strategy documents, but keenly felt by caregivers sensitive to any change in the flow of daily life because of the tight constraints on their time’ (Wright, 2019, p. 348). The robot, therefore, allocated new tasks to the care workers and removed the more emotionally rewarding, autonomous parts of their work, with staff required to mimic the robot during an exercise class rather than leading the session as they had previously done and enjoyed. More recently, a UK local authority has ‘employed’ Pepper, complete with its own staff identity card, and similar concerns about the need to ‘babysit’ the robot in case anything went wrong were raised by its (human) coworkers (Jeffares, 2020).^5^

Another study explored the use of a SAR, ‘Siblot’, in Danish and Finnish care settings (Blond, 2019). The author observed Silbot's hardware and usability problems needed significant intervention from care staff, and software issues required ‘machine babysitters’ to make excuses for some of its ‘inappropriate language’, including loud ‘booing’ when care home residents gave the wrong answer in a quiz. The project manager from one care setting observed: ‘You will always need humans around this system if it has to make sense as well. The system is not capable of delivering the benefits to the world’ (Blond, 2019, p. 122).

These empirical examples of the use of robotics in care settings highlight how technologies alter care worker roles, not by undertaking the more routine tasks (Acemoglu & Restrepo's [2019] productivity effect), instead by encroaching on valued aspects of care work, leaving mundane tasks to care workers and creating new responsibilities, relegating staff to ‘machine babysitters’.

## METHODS

This paper draws on empirical evidence from two studies of technology and care conducted in the UK to examine the new roles and tasks technologies create, and the implications for job quality. In the first part of the results section, we use data from the ‘Advancing Knowledge of Telecare for Independence and Vitality in later lifE’ (AKTIVE) project, presenting findings related to a longitudinal, mixed‐methods (ethnography, interviews, diaries, photography) study with 60 older people (aged 65+ with memory problems and/or susceptibility to falls) and their caring networks (paid and unpaid carers) in two research sites in England. Participants were visited 4–6 times over 6–9 months in their own homes. Fieldwork also included observations at an alarm receiving centre (ARC), a response service and of telecare assessment, installation and reassessment visits (Hamblin et al., 2017).

In the second part, we draw on data from another project (‘Achieving Sustainability in Care Systems: The Potential of Technology’, part of the Sustainable Care research programme) including 40 stakeholder single and group interviews conducted in two rounds (spring 2020 and winter 2020) with 38 participants from: the homecare sector (six participants in nine interviews); technology‐enabled care sector (nine participants in twelve interviews); local authorities (commissioners/technology‐enabled care services [TECS] managers) (12 participants in 13 interviews); and people using adult social care services and carers, and their representative groups (11 participants in 6 interviews). We also conducted eight case studies of the use of technology in adult social care in different local authorities, consulting key members of staff and reviewing publicly available documents including strategies for adult social care, older people, assistive technology, carers, digital; statement of accounts; market position statements; and minutes of scrutiny/other committees and news articles and publications where relevant to technology.

Data from the two projects were analysed using a hierarchical coding frame, with codes related to the research questions, that is, the displacement, productivity or reinstatement effects and the implications for job quality. Data from each study were triangulated using a complex approach of method, investigator and data triangulation (Denzin, 2009). The findings were cross‐checked with empirical studies from the wider academic literature, which was more challenging for the second project focused on an emergent area of policy and practice, with a correspondingly nascent evidence base.

The two examples we explore represent a narrative of the way technology has been used in UK adult social care, starting ‘at the beginning’ with telecare systems, as these were (and still are) the dominant model used in UK adult social care, before turning to the use of mainstream ‘smart’ digital technologies as a developing area of practice. Telecare systems emerged with the use of ‘pull cord’ and user‐worn ‘pendent alarm’ telecare in sheltered accommodation schemes in the 1960s, progressing to include devices to monitor home environments and detect changes which could indicate an emergency (e.g., smoke, flood, temperature extremes) (Doughty et al., 1996). When activated, these devices signal a response unit via radio frequency; the unit then ‘calls’ an ARC using the analogue telephone line; ARCs variously send a response team, call a named responder or the emergency services, depending on the situation and the way the service is organised (Doughty et al., 1996). Telecare is now a subset of a broader range of devices and services—‘technology enabled care services’—that also include telemedicine and telehealth (NHS, 2015). Of the 1.7 million people receiving TECS in the United Kingdom, 1.4 million use pendant alarms (Sugarhood et al., 2014). Analogue‐based TECS in the United Kingdom will need to be decommissioned and replaced by digital alternatives due to the impending—though in some areas already completed—digital switchover (Hamblin, 2020b). However, digital versions of TEC devices are expensive and it is projected replacing analogue devices will cost UK local authorities £150–£300 million (TSA, 2017). In 2020, it was estimated there were 286 million internet‐enabled devices in the United Kingdom (10.3 per household), having increased by 26% in the previous 3 years (AVIVA, 2020) and as a reflection of their ubiquity^6^ and a pressing need to decommission analogue services, attention has turned at both national (DHSC, 2021; Wright, 2020) and local policy levels (Cruickshank & Trim, 2019; TSA, 2017; Wright, 2021a) to the opportunities afforded to adult social care by mainstream ‘smart’ digital devices.

## RESULTS

### Telecare

Using data from the AKTIVE project and the wider literature, we explore the roles telecare reinstates in the UK care system^7^ as though there are telecare *devices*, they require *services* to ‘work’, comprised of assessors, installers, staff in ARCs and ‘emergency responders’. In the AKTIVE project fieldwork, it was rare to observe or hear about a telecare device suffering from a mechanical failure (i.e., when triggered, did not alert an ARC) and yet often users reported dissatisfaction that the telecare had not ‘worked’ as the reply from the ARC or response services were lacking or unexpected (Hamblin, 2017). We explored the human factors which contribute to the way telecare devices perform (Buckle, 2014), underscoring the centrality of these services in the way devices function, and how they reinstate new care roles. As Milligan et al. (2011, p. 347) argue, telecare devices ‘re‐order the place of care‐work and responsibilities to care as new actors become enrolled within the care network and existing care‐givers take on differing roles and responsibilities’ and other authors have also emphasised the reinstatement of new roles—including telecare assessors and installers, monitoring centre and response staff—and how though those working in these jobs ‘co‐produce’ care, they are neglected and underappreciated by both the designers of technologies and services within which they are embedded (Procter et al., 2014; Wigfield et al., 2012, 2013).

Turning first to ARCs, those working in these settings have rarely been invited to engage in research, with the technology itself often the focus and treated as a discrete entity (Farshchian et al., 2017). In England alone, there are 158 ARCs, around 72% of which are provided by local authorities and the remaining 28% by housing associations and private and community interest companies (IPC, 2020). Where ARCs have been explored, staff ‘act as the “glue” providing the all‐important link between otherwise fragmented services’ (Procter et al., 2016, p. 79) and coproduce telecare *services* (as opposed to devices) with unpaid carers to provide optimal outcomes for the person being supported. ARC staff have been found to develop ‘workarounds’ for telecare systems' deficits, which could be ad hoc or in some cases resulted in system change to better suit the needs of their clients (ibid.). Those working in ARCs carry out ‘emotional labour’ (Roberts et al., 2012), providing essential social contact to people who are lonely or confused, and reassurance to those in crisis until help arrives. In the AKTIVE fieldwork, we observed ‘close’ (but geographically distant) bonds between some staff and service users who would activate their devices to speak to someone, rather than solely in an emergency.

However, as with other areas of the care sector, the quality of roles in ARCs has been found lacking in ways that relate to the aforementioned frameworks of ‘good jobs’ and decent work, including precarious employment contracts, emotionally demanding work and verbal abuse (Roberts et al., 2012). It is argued those working in ARCs are ‘archetypal of the contemporary labour market’ as they ‘can be located “anywhere”, require little training, often work to highly controlled practice protocols, and who are time‐managed through computerised performance monitoring and call recording, they can be relatively easily globally outsourced and are usually poorly paid. Their jobs are precarious, yet demanding’ (ibid: 494). ARCs operate as call centres, which have been characterised as ‘repetitive, intensive, often acutely stressful… and that workers' output and performance can potentially be measured and monitored to an unprecedented degree’ (Bain & Taylor, 2000, p. 17).

Those conducting telecare assessment and installations are also under researched (Farshchian et al., 2017) but increasingly important in ensuring devices work reliably during the digital switchover (TSA, 2021b). Procter et al. (2016) highlight how installation staff also ‘co‐produce’ telecare systems with clients and their caring networks, acting in mediatory roles to draw in peripheral but local members of the latter in a formal capacity as emergency responders, thereby ‘strengthening weak ties’ (Yeandle, 2014; c.f. Wilson et al., 2017). We found in the AKTIVE project when information was poorly communicated at telecare's assessment and installation, there were implications for the later use of these devices and outcomes reported by people receiving these services (Hamblin, 2017). For example, it was quite common for users of pendant alarms to report that these devices were not waterproof and could not be worn in the shower or bath, and we observed installation staff relaying this information; however, manufacturers were keen to stress how these devices could be submerged in water for extended periods with no ill‐effects and that they *should* be worn during ‘high risk’ activities such as bathing. We also conducted observations of assessment and installation staff training, primarily delivered by equipment manufacturers which was ‘fragmented and idiosyncratic and may depend on the locality in which the service is provided’ (Buckle, 2014, p. 20).

Telecare emergency response services too have been the focus of limited research, even though those working as ‘first responders’ perform ‘embodied care’ and ‘body work’, attending vulnerable people at times of crisis. Over time, the support required of responders has become increasingly medicalised, initially replacing on‐site wardens in sheltered accommodation who provided out‐of‐hours support with job roles that now include the need for first aid training (Fisk et al., 2020). Response services are not minor players in telecare systems, with just over half of local authorities surveyed (*n* = 79; Steils et al., 2020) commissioning their own instead of relying on unpaid carers or the emergency services. Just as recent research found unpaid carers are treated as ‘resources’ whose involvement is taken for granted when they provide the response for telecare services (ibid.), paid responders too are underappreciated within the design of telecare services and products (Stirling & Burgess, 2020). Even as telecare services become increasingly predictive, highlighting issues before crisis is reached, technology alone cannot act on data generated by monitoring devices—a human response will instead be required before the moment of an emergency (ibid.).

The lack of focus on the roles telecare reinstates reflects a technological bias: the technology is the innovation and though it requires a very human response to function fully, this is overlooked. The relative dearth of literature could also reflect the way these roles are viewed as ‘care‐adjacent’ or separate from care systems and the care workforce as some, such as ARC staff, may never meet the people they ‘care’ for in person though assessors, installers and emergency responders will have face‐to‐face contact with service users. Grisot et al. (2019) argue telecare and data it produces can be used to deliver personalised care and to do so, interpretation and action are required, yet frame this as ‘data work’, distinct from ‘care work’. However, Milligan et al. (2011, p. 353) highlight how technology in care has implications for the boundaries between care settings, shifting from institutionalised settings to home‐based ‘extitutional ones… in which new actors in places remote from traditional care settings are drawn into the care network [and] new care relationships that operate within the home and across both virtual and physical space’. Milligan et al. argue the demarcation between home and more formalised care settings are made porous by technology, and we would argue so too are the boundaries between care and other kinds of supportive work. Roberts et al. (2012, p. 490) argue ‘telecare is not “disembodied” work, but a form of care performed through the use of voice, knowledge sharing and emotional labour or self‐management’; telecare services, including ARCs, are part of ‘care practice’ and therefore shift the definition of care as ‘hands on’ or ‘body work’ (Twigg et al., 2011) to include those in supportive roles facilitated by technology (Milligan et al., 2011; Pols, 2010; Pols & Willems, 2011; Roberts et al., 2012). The failure to recognise those working in telecare services as integral to care work risks casting them as ‘fauxtomatons’ or ‘invisible labour that keeps up the façade of shiny technological efficiency’ (as per Raghav's cartoon).

This inattention has implications for those working in these invisible, yet important, roles. It was apparent that Roberts et al.'s (2012, p. 503) assertion ‘the work undertaken in call centres is often invisible in policy or managerial discussion of telecare’ was still applicable throughout the COVID‐19 pandemic. In the United Kingdom during the pandemic's initial stages, there was a 35% reduction in ARC staffing levels while at the same time, some of those working in these services were retrained and redeployed to other frontline roles such as supporting discharge from hospital. Those already working in response roles received further training to be able to support ‘non‐injured fallers’ in place of emergency services (IPC, 2020; TSA, 2021a), reflecting how the use of technology in care can result in a downward cascade of care work and responsibilities (Milligan et al., 2011). In these difficult times and despite the increasingly integral roles telecare staff were undertaking, the industry's representative body, the Technology‐Enabled Care Services Association (TSA), had to lobby government and liaise with the Department of Health and Social Care to ensure these staff were classed as ‘key workers’ due to the implications for child care, vaccinations and other practical priority measures^8^ (personal communication). The TSA stated ‘the TEC workforce plays a crucial role, 365/24/7 in keeping vulnerable people safe and they need to be recognised alongside other health and care professionals as key workers’ (TSA, 2021a, p. 7). If these roles are rendered invisible ‘fauxtomatons’, there are real implications for those working in these jobs.

## MAINSTREAM TECHNOLOGIES

In our project ‘Achieving Sustainability in Care Systems’ project, we found that local authority adult social care departments were exploring the potential of digital technologies, including mainstream devices such as voice‐controlled virtual assistants and ‘smart’ speakers (e.g., Amazon's Alexa, Echo and Dot; Google's Assistant and Home), wearables such as ‘smart watches’, and other ‘Internet of Things’ devices. The COVID‐19 pandemic was argued by many stakeholders to have accelerated the use of technology in adult social care, including mainstream devices, partly by necessity as some face‐to‐face services were paused. These mainstream devices were discussed in positive terms by all stakeholder groups as being comparatively easy to use and more aesthetically pleasing than traditional TEC devices, as well as being inexpensive when contrasted with specialist products. One technology expert explained:

care technology, often seen as a somewhat intrusive and stigmatising—beige, box and a button, old people's sort of type of intervention—has evolved enormously. And now it can be something fairly familiar…. It could be something as simple as a mobile phone…an example of the future of the care technology, where people will not be given devices‐ we will not be looking at cameras or sensors or physical devices around you. A system will be built into your every‐day life, with devices that you use such as your sockets, your mobile phone, and maybe your watch, maybe your clothing, that will be able to detect any changes, and the information will be shared in a way, in an appropriate way to ensure that you have enough time to adjust your lifestyle. (Technology stakeholder 2, round 1)

Voice‐activated smart speakers in particular emerged from our stakeholder interviews as a new area of interest for local authority commissioners of UK adult social care services (Hamblin, 2020b; Wright, 2021a). Our stakeholder interviews, case studies and policy reviews identified widespread pilots of smart speakers in adult social care amongst local authorities, some utilising national funding opportunities. Of our local authority case studies, one in particular had been the ‘vanguard’ in terms of the use of mainstream technologies, having been the first to trial smart speakers in adult social care using national programme funding. A pilot of 50 people with the aim of providing more personalised care services while also reducing short ‘in‐person’ care visits and their associated costs was deemed a success, with the council reporting savings related to the trial participants of in excess of £66,000. Buoyed by this, the local authority had entered into a partnership with a ‘technology agnostic’ brokerage service and were used as an exemplar across the local government and technology sectors.

There were other examples of local authorities using ‘smart’, voice‐activated speakers as a way to provide information to residents about local services but also to give users greater control of their home environments (changing lighting, temperature, setting reminders). Local authorities were either using existing ‘skills’ (a voice‐activated app) not specific to care to—for example, turn off the lights or play music—or were working with technology providers to develop bespoke skills (including medication reminders; a messaging system for people receiving care and their carers; a directory of ‘trusted’ service providers; and a system to record care tasks). There were also a few examples of local authorities who were considering replacing their telecare services with smart speakers. However, this neglects an important difference in the way they are configured: both telecare devices and smart speakers can be arranged to make a telephone call to another person but the former are typically programmed to call an ARC first, who then contact a responder/s if the user does not reply or indicates they need help. This additional step ensures that the user is not left in need of support if the link between the device and responder technically functions, but the responder does not answer their telephone. Currently, no smart speakers have been connected to an ARC and one local authority commissioner noted should this happen, the entire TECS market would collapse.

The local authority commissioners were keen to explore the potential cost savings mainstream devices could generate. However, the commissioner from the ‘vanguard’ case study that first trialled these devices emphasised caution, noting that mainstream technologies, although seemingly cheap, ‘standalone’ and completely ‘disembodied’, if provided as part of adult social care would still need to be installed and maintained. As such, the commissioner argued there was:

a complete underestimation of the amount of effort that's required to make it happen. And I think that's the case here, is ‘oh, you know they can use the Amazon Alexa’ and that most people I talk to who say things like that, they've no clue about the challenges… the level of support that's required… There's a reason why that 80‐year‐old granny isn't using the Alexa that their 45‐year‐old grandson bought them and that is that they haven't got a clue what to do with it. Actually they need ongoing support to do that. Who's going provide that?… you've got a role that is just beyond ‘oh use an Alexa’, and then what's the resource implications for that? And so we've shown really clearly the level of support people need on ongoing basis is quite severe. (Local authority stakeholder 11, round 2)

Another local authority commissioner cited an example of where they had worked in partnership with a technology provider to deliver 300 older people wearable smart devices to record information on their heart rates, sleeping patterns, exercise and use of home appliances. They noted the users required a lot of initial and ongoing support to engage with the device and a serious problem was presented by an upgrade to the technology provider's system, which required staff to take all 300 users through the changes required for the technology to continue to function—capacity the local authority simply did not have.

Smart mainstream technologies when used in adult social care, therefore, require human support to maintain their façade of efficiency, and as with the services that enable traditional telecare to function, there is a risk they will be underappreciated ‘fauxtomatons’, servicing these apparently intuitive devices and supporting people to use them. With their invisibility comes the risk that these roles too will perhaps not be ‘good jobs’ or quality work. Also, from a cost perspective, these devices may not present the levels of savings anticipated by commissioners as they either will result in a wasted resource if users are not supported to engage with them, or if commissioned with appropriate ‘wraparound’ services, additional investment will be required. An alternative scenario is that if local authorities do not provide this support, responsibility may fall to unpaid carers, identified as an issue with telecare devices (Steils et al., 2020).

## DISCUSSION: FAUXTOMATONS AND MACHINE BABYSITTERS

This paper has linked the literature on automation, ‘good’ and ‘decent’ work and STS to examine the implications of the use of technologies in adult social care. To return to the research questions, the impact of technology on care sector jobs is more complex than the simple replacement of human labour: technology also reshapes existing roles, and creates new forms of work. At a time when recruitment and retention in the care sector are pressing issues, the quality of these newly created or reinstated roles is an important consideration, as is what tasks technologies leave behind for humans to attend to.

Reflecting on the implications for existing roles, technology *could* reconfigure care jobs—characterised as ‘dirty’, unskilled and undesirable—into good quality, well‐paid and highly esteemed work, as per Acemoglu and Restrepo's (2019) productivity effects. However, the example of the use of robotics in residential care highlights how care worker roles can be ‘degraded’ by technology, shifted from being person‐focused to ‘machine babysitters’, assisting others to engage with purportedly user‐friendly devices or protecting expensive investments from misuse. Technology, therefore, rather than solving the issues related to recruitment and retention in the care sector, could exacerbate them by degrading care work through the removal of its valued aspects and adding new, technology‐focused tasks.

The examples of telecare and digital smart mainstream devices highlight how technology can reinstate new jobs and forms of work in adult social care, bringing new actors into the caring domain, including assessment, installation and monitoring centre staff and emergency responders. These roles are integral to making technology ‘work’ as telecare devices themselves do not answer emergency calls or physically attend and help those in need—reassuring someone while they wait for the response service, helping someone up who has fallen, waiting with them until the emergency services arrive, and in some sad instances, bearing witness to a person's last moments—and yet the policy discourse is technology‐focused, rarely considering the importance of human factors in mediating technologies' successes or failures. Mainstream smart technologies are an area of interest for UK local authority commissioners but these too need *services* to help people use these devices in the first instance as well as process and act on data they may generate. There is a risk that some of the new or reinstated roles created by the use of these digital devices in adult social care could be ‘fauxtomatons’, providing the façade of efficiency and invisibly providing care on behalf of supposedly caring technologies. However, it was apparent some local authorities underestimated—influenced perhaps by the technocentric policy discourse—the need for ‘wraparound’ services in their desire to create innovative and sustainable care. Pilot projects served as valuable lessons as to the importance of ongoing support to ensure devices ‘worked’, but did then raise questions as to whether in practice technology would deliver on promised resource and workforce savings.

The sleight of hand where roles created by technologies are rendered invisible, peripheral, ‘care‐adjacent’ ‘fauxtomatons’ to maintain the façade of technological efficiency has implications for job quality. Returning to the discussions of ‘good’ jobs and quality work, though contested concepts, safety at work and job content are both consistently cited as integral and there are issues related to the demands placed upon those working in these ‘care adjacent’ roles including of stress, emotional labour and abuse. In practice therefore technology in social care could do more to create new, precarious and poor quality forms of work that are ultimately invisible and undervalued than it could to improve workforce capacity and create cost savings.

Just as technology is not asocial, completely independent of human action, it is also not apolitical. The framing in policy discourse of advancements in technology as both a solution to the crisis facing care systems and a natural progression,^9^ separate from and unstoppable by human action, diverts attention from both the possibility of and necessity for alternative strategies for change. Matt Hancock, the former Secretary of State for Health and Social Care, claimed ‘tech transformation is coming’ (Hancock, 2018) but the notion that this is inescapable and indisputably good for all in caring contexts is an example of ‘the supreme and most insidious exercise of power’, persuading people to ‘see [something] as natural and unchangeable’ (Lukes, 2005, p. 28). Rather than answering calls from the care sector and academia (Glasby et al., 2021) to adequately address the fundamental issues that underlie the recruitment crisis in UK adult social care, including the way care is resourced,^10^ organised and ultimately valued, policymakers have presented technology as the solution for an over‐stretched, under‐paid, under‐esteemed workforce. We have highlighted that rather than improve workforce capacity and save resources, technology in social care could degrade existing roles and create new, precarious and poor quality forms of work that are ultimately invisible and undervalued. It is crucial that researchers open the ‘black box’ of technology which increasingly includes human labour to examine the quality of jobs therein and challenge notions of inescapable and wholly beneficial technological advancement in care—and other—sectors.

## ETHICS STATEMENT

The AKTIVE Project received ethical approval from the Social Care Research Ethics Committee (reference number: 11‐IEC08‐0045) and the Oxford Health NHS Foundation Trust (reference number: 95833). The ‘Achieving Sustainability in Care Systems: The potential of technology’ was approved by the University of Sheffield Research Ethics Committee (reference number: 026350), received ADASS endorsement (reference number: RG19‐08), University of Sheffield research governance sponsorship (reference number: 148644) and followed relevant local authority research governance procedures (where applicable).

## ENDNOTES

1 ‘An umbrella term referring to the technical devices and professional services applied in “care at a distance” that address and support people in need of care’ (Pols, 2012, p. 110).

2 Certainly the edict ‘thou shalt upskill!’ (Schlogl et al., 2021, p. 14) is applicable to the care workers, with a policy focus on the skills deficit of a workforce purportedly being left behind by fast‐moving technological developments (DHSC, 2021; NHS & PHE, 2017).

3
https://krishcat.com/

4 Though there is no universal definition of a ‘robot’, a typology used in social care (Consilium Research & Consultancy, 2018) includes (1) physically assistive robots which perform discrete tasks either independently of care workers or alongside in a supportive role; (2) socially assistive robot which are grouped into two further types: ‘service robots’ which assist with activities of daily living, including reminder systems and ‘companion robots’ focused on improving wellbeing; and (3) cognitive assistance robots designed to support people to perform cognitive tasks.

5 Pepper has recently been ‘unplugged’ by its manufacturer (SoftBank) due to poor sales (Bottery, 2021).

6 However, people most likely to use adult social care (those aged over 65 or disabled) are least likely to have an internet connection or use an ‘Internet of Things’ enabled device (ONS, 2020).

7 Other studies have explored telecare's impact on care worker roles (c.f. Højlund & Villadsen, 2020).

8
https://www.tsa-voice.org.uk/-covid-20/updates-guidance

9 ‘Natural’, but paradoxically requiring the stimulation of extensive national funding programmes.

10 The recent introduction (09/21) of a health and social care levy has been critiqued for failing to address fundamental issues in adult social care and prioritising the National Health Service (Care Worker's Charity, 2021; Trade Union Congress, 2021). The long‐awaited White Paper (DHSC, 2021) has been met with a largely critical response from the care sector and academia, described as under‐resourced and ‘merely tinkering’ with a care system in dire need of radical reform and resources (Yeandle, 2021).
